# Synaesthesia-type associations and perceptual changes induced by hypnotic suggestion

**DOI:** 10.1038/s41598-017-16174-y

**Published:** 2017-12-11

**Authors:** Sakari Kallio, Mika Koivisto, Johanna K. Kaakinen

**Affiliations:** 1 School of Bioscience, University of Skövde, Högskolevägen, Box 408, 541 28 Skövde, Sweden; 2 Centre for Cognitive Neuroscience, University of Turku, Assistentinkatu 7, FIN-20014 Turku, Finland; 3 Turku Institute for Advanced Studies and Department of Psychology, University of Turku, Assistentinkatu 7, FIN-20014 Turku, Finland

## Abstract

Are synaesthetic experiences congenital and so hard-wired, or can a functional analogue be created? We induced an equivalent of form-colour synaesthesia using hypnotic suggestions in which symbols in an array (circles, crosses, squares) were suggested always to have a certain colour. In a Stroop type-naming task, three of the four highly hypnotizable participants showed a strong synaesthesia-type association between symbol and colour. This was verified both by their subjective reports and objective eye-movement behaviour. Two resembled a projector- and one an associator-type synaesthete. Participant interviews revealed that subjective experiences differed somewhat from typical (congenital) synaesthesia. Control participants who mimicked the task using cognitive strategies showed a very different response pattern. Overall, the results show that the targeted, preconsciously triggered associations and perceptual changes seen in association with congenital synaesthesia can rapidly be induced by hypnosis. They suggest that each participant’s subjective experience of the task should be carefully evaluated, especially when studying hypnotic hallucinations. Studying such experiences can increase understanding of perception, automaticity, and awareness and open unique opportunities in cognitive neuroscience and consciousness research.

## Introduction

Some highly hypnotizable individuals experience visual hallucinations under hypnotic suggestion^1,2^. Debate is ongoing regarding whether these experiences are based on use of goal-directed mental imagery or if they occur automatically: i.e., without cognitive effort^3–6^. The automaticity of hypnotic visual hallucinations has recently been studied by attempting to induce a condition that would either mimic synaesthesia or temporarily disrupt it^7–9^. In synaesthesia (from the Greek, “sensing together”), ordinary perceptual stimuli such as letters or sounds elicit an automatic additional experience in the same or another perceptual modality^10^.

Synaesthetes are a heterogeneous group: both the stimuli that trigger the synaesthetic experience (the *inducer*:^11^) and the synaesthetic experience itself (the *concurrent*) vary widely. The experience can differ from a vaguely imagined association to actual perception^12^. The most typical example is *grapheme-colour synaesthesia* (GCS) in which a letter or number (i.e., a *grapheme*) generates an association or perception of some colour^12^. A colour can also be associated with a certain note (*chromestesia*) or form (*form-colour synaesthesia*: FCS). About 10% of GCS synaesthetes, referred to as *projectors*, report experiencing the concurrent as a perception in external space, typically above the presented grapheme; whereas the vast majority of synaesthetes, referred to as *associators*, report experiencing the concurrent only in their mind^13^. Importantly, the synaesthetic precepts are never confused with reality^14^.

Hypnotic responses between individuals are likewise heterogeneous. Highly hypnotizable persons experience the same suggestions quite differently. Suggestions aiming to produce changes in visual perception (e.g., hallucinations) can result in very different conscious experiences^4,15–17^ even among individuals with exactly the same score in the most widely used tests measuring hypnotic susceptibility: the Harvard Group Scale of Hypnotic Susceptibility Form A (HGSHS:A)^18^ and the Stanford Hypnotic Susceptibility Scale Form C (SHSS:C)^19^.

The major neurocognitive theories of synaesthesia suggest it to be a congenital condition characterized by changes in cortical connectivity^20^. Some models propose that later information can influence earlier stages of information-processing via feedback, thereby affecting the final conscious experience^11,21^. According to the *feedback model*, synaesthesia-like experiences can be artificially created by training^22^ or hypnosis^8^. In hypnosis research, one finds a comparable model^4,23^ proposing that hypnotically induced colour alterations can occur if suggestions modulate the recurrent interactions^4^ that generate the final conscious perception. Meanwhile, sociocognitive theories argue that all hypnotically induced visual experiences are the result of goal-directed mental imagery^24^ and can equally well be experienced without hypnosis.

A central criterion for synaesthetic experience is that it should happen automatically: i.e., without any wilful effort to produce the concurrent experience^10,25^. The same criterion has been proposed for hypnotic behaviour and perceptual changes^6,26–28^. Evidence suggests that awareness of the inducer shape is necessary both in GRC and hypnotic colour hallucinations for the colour experience to emerge^4,29^. Sociocognitive theories argue that perceptual changes always result from the use of mental imagery and that experienced involuntariness is triggered by situational, self-generated events (e.g.^27^); while the Altered State Theory of hypnosis (AST) (see also^26,28,30–34^) argues that hypnotically induced changes in perception can be triggered preconsciously and so automatically.

The hypnotic suggestion to induce a functional analogue of GCS or FCS hypnotically could be something like “when you see a square on the screen, it will always be red”. A highly hypnotizable person should then develop a strong association of all targeted inducers (squares) being red – or, in some cases, even see them as red. Sociocognitive theories claim that this experience is not similar to synaesthesia and that conscious recognition of the target inducer (as a square) is first required, followed by cognitive effort to imagine it being red. In contrast, AST predicts that an automatic association emerges and that some highly hypnotizable individuals should even be able to perceive the inducer and concurrent simultaneously, just as some genuine synaesthetes^23,26^. Simultaneous synaesthetic experience should make naming the targeted symbol (square) “red” just as easy, requiring the same time and involving the same eye behaviour as naming a non-targeted symbol by its veridical colour. Cognitive efforts to imagine the colour mentally – required by sociocognitive theories – or simulate synaesthetic behaviour by recalling the inducer/concurrent association should slow down the naming of targeted symbols by the suggested colour, since the veridical colour of the inducer should interfere. Fixation times on targeted symbols should likewise increase.

The present study examined whether hypnotic suggestion can induce experiences resembling congenital FCS. We used various symbols in a modified “synaesthetic Stroop” paradigm^35^. The participants had to name the colour of symbols while three of the symbols were hypnotically suggested to bring about an inducer/concurrent synaesthetic experience. We used symbols as inducers instead of graphemes to make the stimuli more clearly distinctive. Two previous studies came to contradictory conclusions whether^8^ or not^7^ hypnotically induced GCS experiences occur automatically. This study has important methodological advantages over either study. First, we increased the level of difficulty by using three inducer/concurrent pairs that needed to be remembered explicitly throughout the naming process, ensuring heavy cognitive load unless the concurrent was experienced automatically. Second, we used high-resolution eye tracking, making it possible to evaluate cognitive effort and strategy for each stimulus. Third, we videotaped all sessions so that afterwards participants could watch their own performance during the experimental condition. We then interviewed participants concerning strategies used and how stimuli were experienced subjectively^17^. Fourth, we used two hypnosis groups: a test group of highly hypnotizable individuals (“*High*” Group: HG) and a control group of low (weakly) hypnotizable individuals (Control “*Low*” Group: ConLoG); plus a second control group, who were instructed to simulate the behaviour of persons experiencing a colour hallucination (Control Simulators Group: ConSimG). Both types of controls are commonly used in hypnosis experiments^15,36^, but seldom in one and the same experiment.

If hypnotic suggestions can create an automatic association between hypnotically suggested inducer and concurrent, this would support AST and the view that synaesthesia-type experiences can exist without abnormal neural connections. An automatic process accompanied by changed colour experience would make naming inducer symbols by the suggested concurrent colours easier and faster compared to cognitive strategies such as recalling from memory. At the same time, it would hamper naming the veridical colour of the inducer when in conflict with the hypnotically suggested concurrent^25^. Since previous research indicates that even highly hypnotizable individuals may experience the task quite differently^4,15–17^, we designed the experiment in such a way that the results need not be analysed at the group level but instead on a single-trial basis: i.e., separately for each participant. We present the results for each highly and low (weakly) hypnotizable participant separately. We expect that only some members of the HG may experience vivid visual hallucinations and thus resemble the projector-type synaesthetes phenomenally and behaviourally. Others should resemble associators, experiencing the concurrent only as an association in the mind.

## Results

The experiment had three conditions, all with instructions to say the veridical colour of symbols presented on a screen. The experiment started and ended with a normal condition (NC and NC2). Between these was an experimental condition (EC) where the task was preceded by hypnotic suggestions intended to bring about a synaesthesia-type colour change in three of the symbols.

Table 1 presents the mean trial times and number of errors in the different task conditions for each participant in the HG (*n* = 4).

**Table 1:** Trial times (observed means, standard deviations, regression coefficients and their 95% confidence intervals) for the HG, plus number of cases of synaesthesia and erroneous responses under each condition. *B* = unstandardized regression coefficient for comparison between tasks using NC as the baseline. *LL* = lower limit of the 95% confidence interval (CI) for the regression coefficient. *UL* = upper limit of the 95% CI. A synaesthesia suggestion was given for 78 targets. There was a total of three trials and 75 items for NC and NC2, six trials and 150 items for EC.

Participant	Task	Trial time						
		M	SD	B	LL	UL	Synaesthesia	Errors
S1	NC	24.69	0.49					0
	EC	24.31	1.42	−0.38	−2.21	1.44	77	0
	NC2	24.91	0.77	0.22	−1.89	2.33		0
S2	NC	16.59	3.31					2
	EC	149.69	21.81	133.10	106.96	159.23	8	4
	NC2	14.64	0.65	−1.95	−32.13	28.23		3
S3	NC	15.13	3.41					0
	EC	28.41	2.80	13.28	8.70	17.86	68	7
	NC2	18.93	2.38	3.80	−1.49	9.08		0
S4	NC	20.85	0.86					3
	EC	34.11	4.33	13.25	7.97	18.53	3	9
	NC2	17.96	1.16	−2.90	−8.99	3.20		0

**Figure 1:**
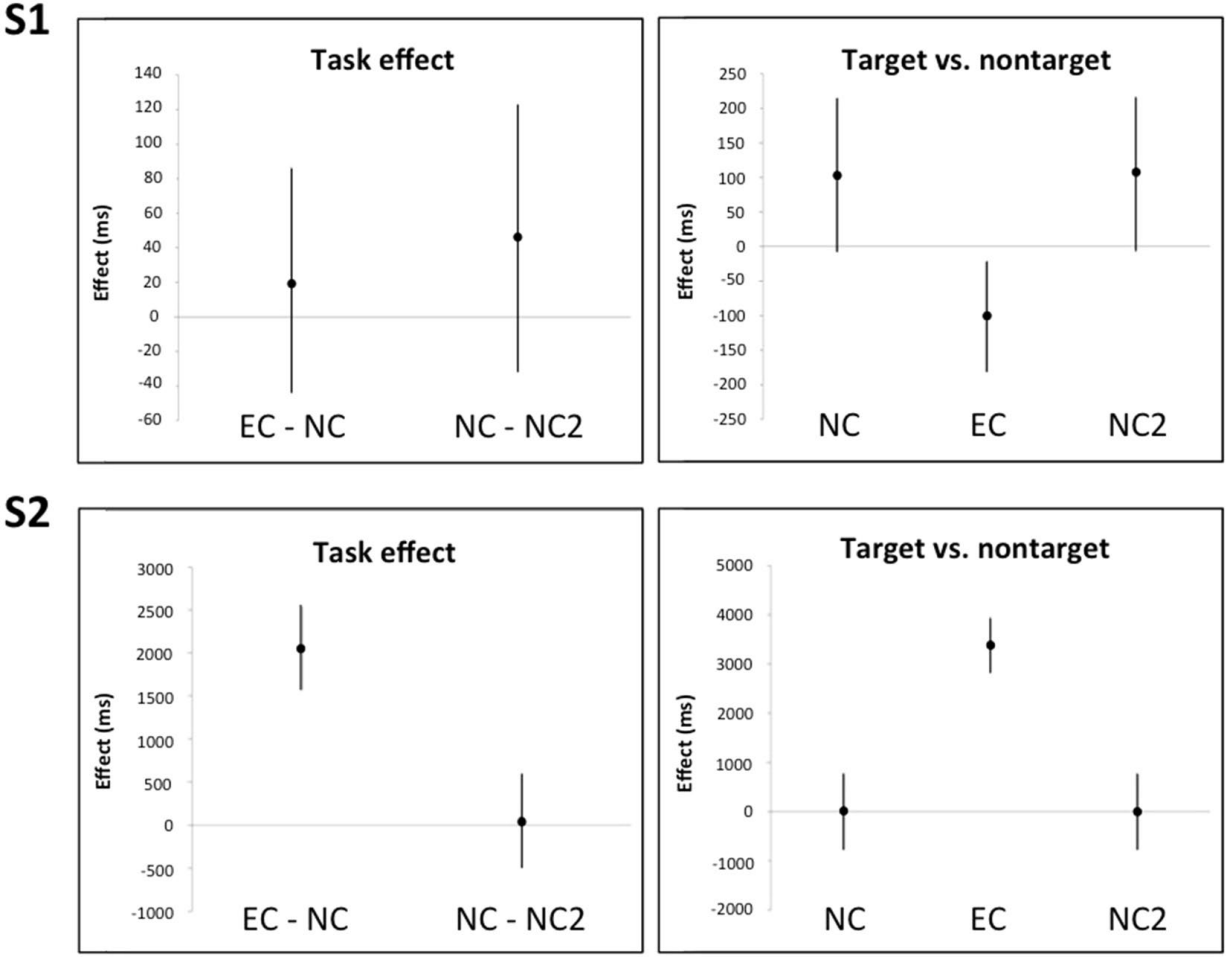
Model estimates (error bars represent 95% CIs) for task effects (left panels), and difference between targets and non-targets in NC, NC2, and EC (right panels) for participants S1 and S2.

As the table shows, S1 made no errors under any condition and produced synaesthetic effects for all but one target (77). Mean time spent on a trial did not depend on task, neither were there reliable differences between tasks in mean gaze durations. However, in EC, S1 showed shorter gaze durations toward targets than non-targets, with the inverse in NC and NC2 (Fig. 1).

In the post-experiment interview, S1 did not remember receiving any suggestions: i.e., S1 experienced posthypnotic amnesia. S1 could not believe that she made so many “mistakes” when shown the EC video and wondered if the video could be real. S1 was puzzled when asked about strategy since she experienced each condition as being the same.

***S2*** made five errors between NC and NC2, and produced synaesthetic effects for eight targets in addition to making four errors in EC (Table 1). The synaesthesia cases were shapes for which S2 initially named the suggested colour, even though she always corrected her initial impression and named the actual colour subsequently. S2 showed a clear tendency to slow down in EC in comparison to naming tasks NC and NC2. Analysis of gaze durations revealed that this slow down was reflected in eye movements (Fig. 1): i.e., in EC S2 showed remarkably longer gaze duration towards targets relative to non-targets (more than three seconds longer on average), whereas in NC and NC2, gaze duration showed no such influence.

S2 remembered receiving suggestions but not what they were about. “I felt a strong urge to name aloud a colour but then I saw that it was not the right colour. The strategy was to try to avoid naming the colour that I felt was correct, but to try to say the colour I saw with my eyes”. The significant slowing down of gaze duration towards targets is in line with S2’s subjective reports.

**Figure 2:**
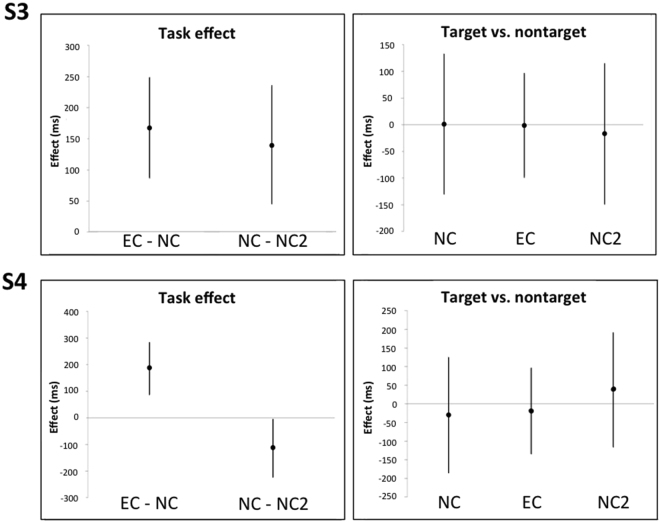
Model estimates (error bars represent 95% CIs) for task effects (left panels), and difference between targets and non-targets in NC, NC2, and EC (right panels), for participant S3 and S4.

S3 made no errors in NC or NC2. She produced synaesthetic effects for 68 targets and made seven errors in EC. Her trial times were longer in EC than in NC and NC2, as was her gaze duration; however, her gaze duration did not vary between target and non-target stimuli under any condition (Fig. 2). S3 reported perceiving crosses always according to the hypnotic suggestion in EC. We carried out further analysis, comparing gaze duration towards crosses vs. other targets under the different conditions. Gaze duration did not differ under either of the “normal” naming conditions (NC1: *b* = 103.38 [−88.45, 295.22], *t* = 1.07; NC2: *b* = −12.51 [−210.85, 185.82], t = −0.125), whereas in EC gaze duration was 210.15ms faster [−348.19, −72.11, *t* = −3.01] towards crosses than other targets. Gaze duration toward crosses neither slowed down nor sped up in EC compared to NC and NC2 (NC1: *b* = −37.69 [−229.53, 154.15], *t* = −0.039; NC2: *b* = −90.55 [−287.65, 106.54], *t* = −0.91).

S3 remembered the suggestions. “I saw the crosses as blue and they were easy to name; however, squares and circles were more difficult since I was not always sure what the correct colour was. I did not see the colour of squares and circles on the screen as suggested like I saw the crosses. I had no strategy to try to memorize the suggested colours”. The differences in gaze duration in EC are in line with S3’s subjective reports.

**S4** made three errors in NC (none in NC2) and produced synaesthetic effects for three targets while making nine errors in EC. Her trial times were longer in EC than NC and NC2. Gaze duration was longer in EC than in NC and NC2 but shorter in NC2 than NC, gaze duration did not depend on target type under any condition (Fig. 2).

S4 said: “I remembered the suggestions but I did not want to follow them; however, it was difficult to come up with the name of the correct colour which I saw that the symbols had”.

Table 2 presents the mean trial times and number of errors under the different task conditions for each participant in the low hypnotisability control group ConLoG (*n* = 4).

**Table 2:** Trial times (observed means, standard deviations, regression coefficients and their 95% CIs), plus number of cases of synaesthesia and erroneous responses under each condition for ConLoG. *B* = unstandardized regression coefficient for comparison between tasks using NC task as the baseline. *LL* = lower limit of the 95% CI for the regression coefficient. *UL* = upper limit of the 95% CI for the regression coefficient. A synaesthesia suggestion was given for 78 targets. There was a total of three trials and 75 items for NC and NC2, six trials and 150 items for EC.

Participant	Task	Trial time						
		M	SD	B	LL	UL	Synaesthesia	Errors
S5	NC	14.46	0.21					0
	EC	15.69	0.73	1.23	0.237	2.23	0	1
	NC2	13.22	0.61	−1.24	−2.39	−0.09		0
S6	NC	15.29	1.13					0
	EC	18.20	1.23	2.92	1.00	4.83	0	4
	NC2	18.24	1.19	2.95	0.74	5.17		1
S7	NC	16.98	1.15					0
	EC	28.52	3.26	11.54	7.41	15.66	0	0
	NC2	16.35	1.40	−0.63	−5.39	4.13		1
S8	NC	12.20	0.11					0
	EC	16.05	0.76	3.85	2.92	4.78	0	0
	NC2	11.92	0.25	−0.28	−1.35	0.79		0

In EC, each participant in ConLoG named the colours according to their true wavelength. One participant asked if the purpose was to try to name the true colour or if she should try to follow the suggestion. The instructions – to name what she saw on the screen – was repeated to her. Interviewed afterwards, nobody reported using strategies other than simply completing the task. No one reported attempting to memorize the instructions.

**Figure 3:**
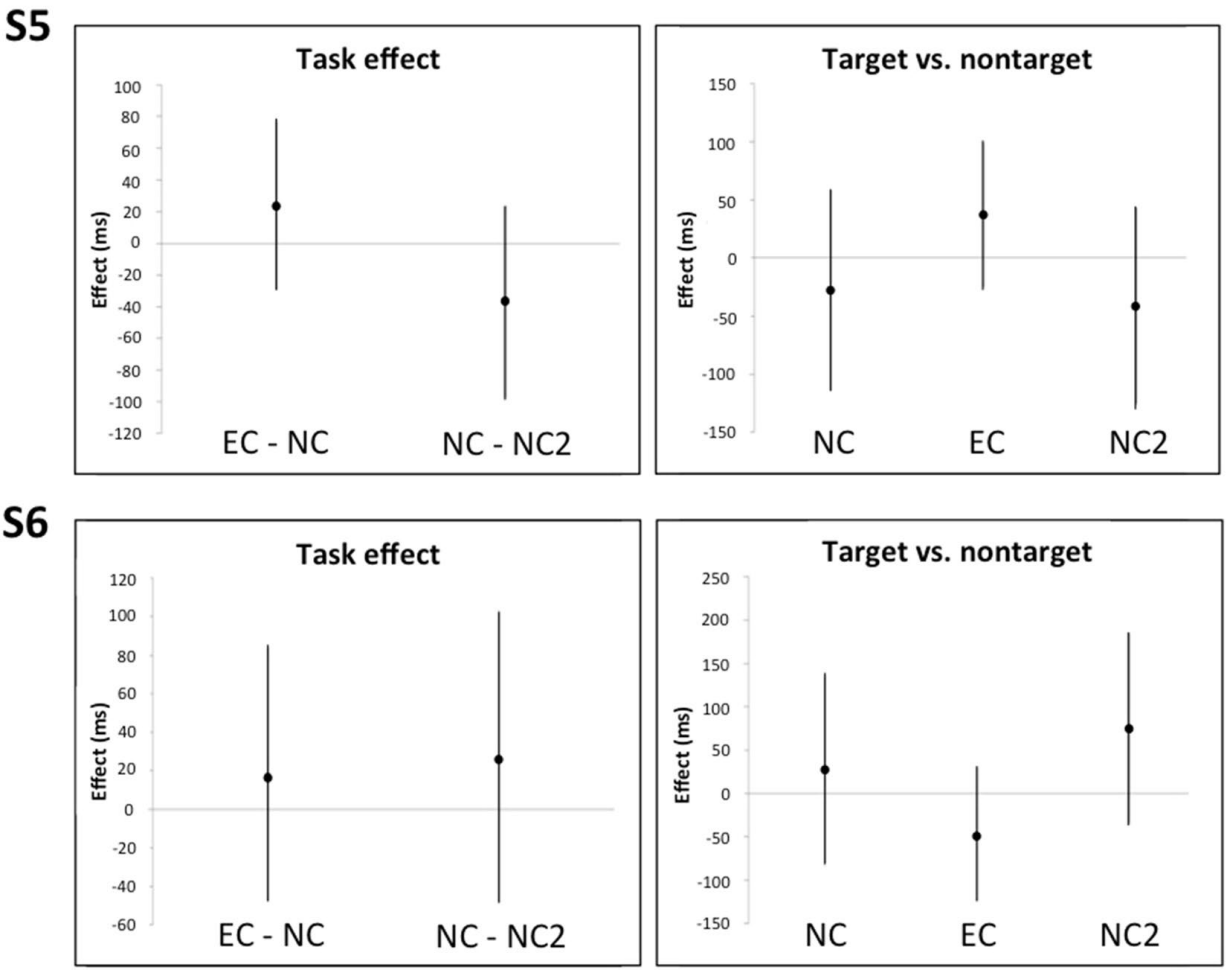
Model estimates (error bars represent 95% CIs) for task effects (left panels), and difference between targets and non-targets in NC, NC2, and EC (right panels), for participants S5 and S6.

**S5** made no errors in NC or NC2 and only one in EC. Trial times were longer in EC than NC and NC2, and shorter in NC2 than NC (Table 2). Gaze duration did not reveal any clear effects of task or item type (Fig. 3).

**S6** made one error in NC and four in EC. Trial times were longer in EC and NC2 than NC. Gaze duration did not reveal any clear patterns (Fig. 3).

**Figure 4:**
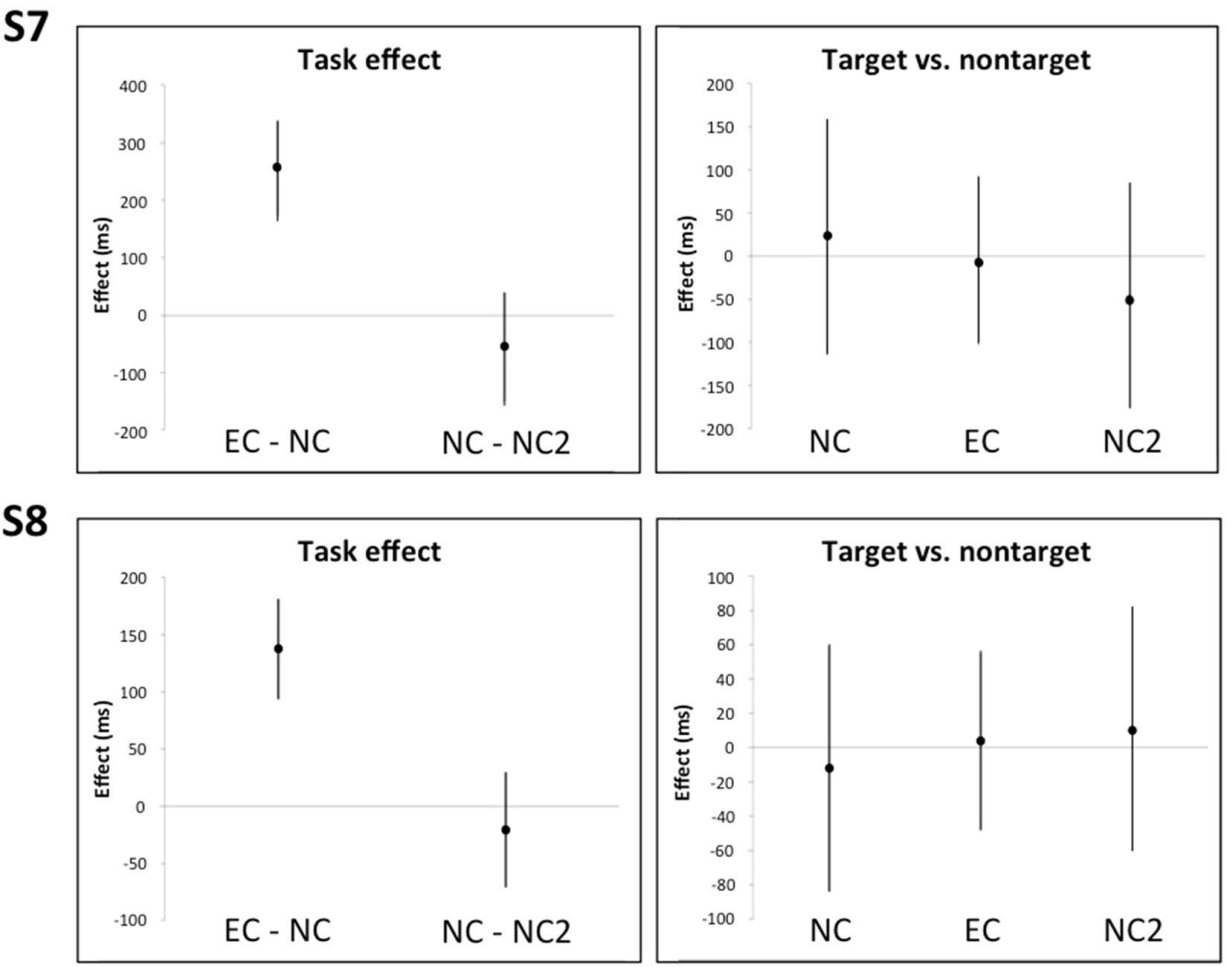
Model estimates (error bars represent 95% CIs) for task effects (left panels), and difference between targets and non-targets in NC, NC2, and EC (right panels), for participants S7 and S8.

**S7** made one error in NC and none in the other tasks. Trial times and gaze duration were both longer in EC than NC and NC2. There were no clear effects of item type under any condition (Fig. 4).

**S8** made no errors under any condition. Trial times were longer in EC than NC and NC2. Gaze duration was longer in EC than NC and NC2; there were no reliable differences between targets and non-targets in any of the tasks (Fig. 4).

Three participants in the simulator control group ConSimG were left out from analysis: a substantial portion of eye movement data in EC was lost due to head movements. One participant said it was difficult to remain still given the task difficulty. Analysis was done with the remaining five participants.

**Figure 5:**
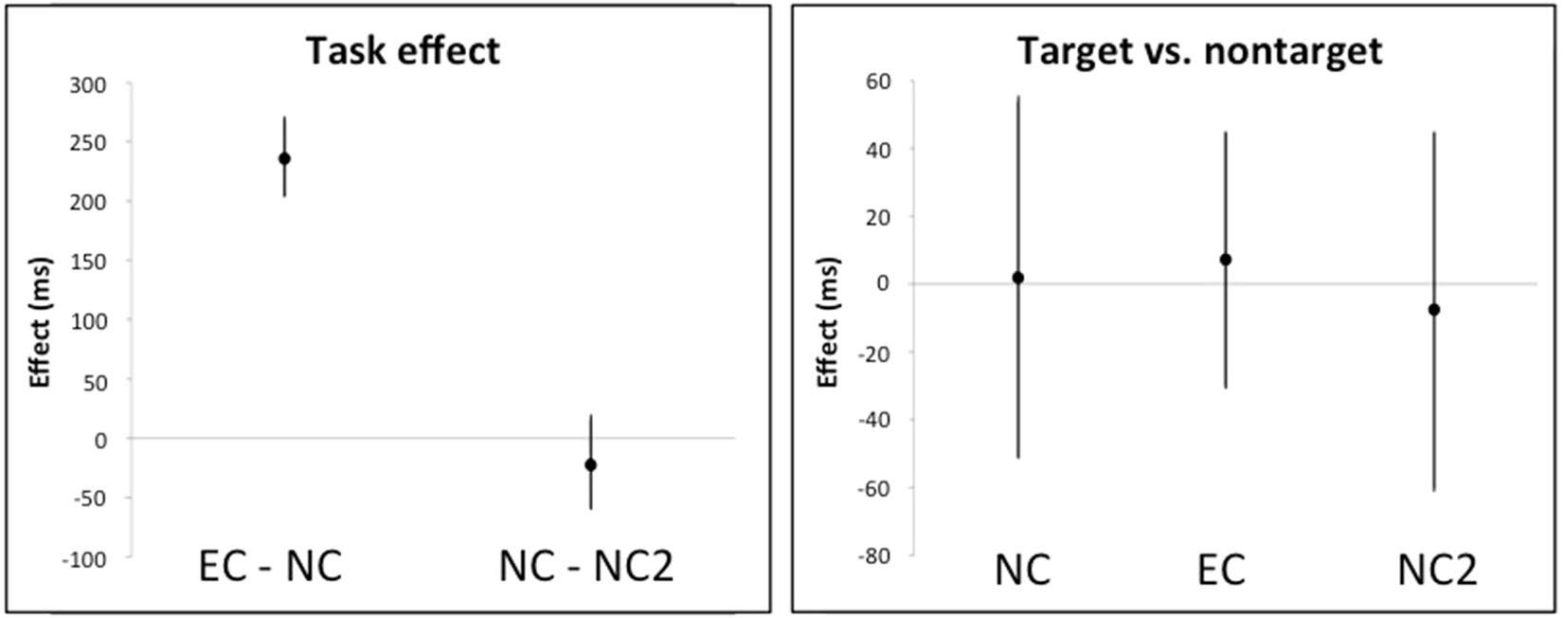
Model estimates (error bars represent 95% CIs) for task effects (left panel), and difference between targets and non-targets during NC, NC2, and EC (right panel) for ConSimG.

Table 3 presents the mean trial times and number of errors under the different conditions for each participant in ConSimG (*n* = 5). Overall, they produced an average of 0.40 errors (SD = 0.89) in the naming task (all in NC2) and 1.40 in EC (SD = 1.14), with 76.80 synaesthetic effects (SD = 0.45). They showed a clear slow down in EC compared to the naming tasks, which was reflected in longer gaze durations (Fig. 5). There were no reliable differences in gaze duration between targets and non-targets.

**Table 3:** Mean trial times (observed means, standard deviations, regression coefficient and its 95% CI), plus mean number of cases of synaesthesia and erroneous responses under each condition (standard deviations in parentheses) for ConSimG (*n* = 5). *B* = unstandardized regression coefficient for comparison between tasks using NC as baseline. *LL* = lower limit of the 95% CI for the regression coefficient. *UL* = upper limit of the 95% CI for the regression coefficient. A synaesthesia suggestion was given for 78 targets. There was a total of three trials and 75 items for NC and NC2, six trials and 150 items for EC.

Task	Trial time						
	M	SD	B	LL	UL	Synaesthesia	Errors
NC	14.95	1.28					0(0)
EC	21.64	2.44	6.69	5.63	7.75	76.80 (0.45)	1.40 (1.14)
NC2	13.99	1.18	−0.96	−2.18	0.26		0.40 (0.89)

ConSimG participants reported these strategies:

I tried just to remember the instructions. I repeated them in my mind in order to remember them.

I tried to visually memorize the image where all the three symbols were shown. That helped me to remember the symbols that had to be named differently.

I tried to remember the image where the symbols were presented but in the third round I already had them in my memory and then the task became easier.

I tried to remember the image with the symbols in order to keep them in my mind. I thought that the circle was like a red ball so that was easiest to remember.

I tried to memorize the symbols when I saw them. I tried to make some rules but they were not that effective: e.g., the *risti* (“cross”) has a letter “s” in it and the word *sininen* (“blue”) starts with the letter “s”. The green square reminded me of a football pitch or a golf green.

A clear difference can be seen between HG and the two control groups (ConLoG, ConSimG) in how participants interpreted the task. ConSimG was quite homogenous: each participant did the task the same way, used similar strategies for remembering the three inducer/concurrent associations, used approximately the same time in NC and NC2, was clearly slower in EC, and showed no target effect in eye behaviour. ConLoG was likewise homogenous and consistently (somewhat) slower in EC. No one followed the hypnotic suggestion, instead naming all symbols in all conditions according to veridical colour: i.e., performing the same task three times. The suggestion of three symbols always being a certain colour did not result in specific difficulties with these symbols. Rather, it led to a slow down when naming the symbol colours in general. No one noted experiencing any changes in how they saw the colours.

HG did not perform EC at all homogenously. Two participants (S1, S3) named the colours for the target objects (and stated seeing them, or some of them) according to the hypnotic suggestion. The other two participants (S2, S4) named the veridical colours of all objects and did not note seeing them otherwise (i.e., per the suggestion).

Three members of HG (S1, S2, S3) showed a target effect. Analysis of the eye movements supports the statements of the two participants (S1, S3) who claimed seeing all (or some) of the targets *per* the suggestion. These targets were processed as fast or slightly faster in EC compared to NC/NC2. S2 used a different strategy, naming the veridical colours in EC. The hypnotic suggestion led to clear interference in the naming process.

The members of HG differed as well between NC and NC2. The two participants (S2, S4) who named the veridical colours in EC were somewhat faster in NC2 than NC. One participant (S1) who followed the hypnotic suggestions had exactly the same speed in NC and NC2 while the other (S3) was clearly faster in NC.

Every participant accepted a post-experiment challenge with a prize of €50. Their task was to simulate a hallucinator and repeat the EC, trying to get a faster time than in NC – this time with hints for remembering the right colours for the three target symbols. Due to the very different strategies they used in the experiment, some participants had a clear advantage. Indeed, a few said they had already memorized the critical symbol/colour combinations; however, most participants still found the hints helpful. Nevertheless, no participant collected the reward.

## Discussion

Our results clearly show that hypnotic suggestion can generate a condition functionally similar to synaesthesia – as discussed recently by e.g.^10,37,38^. There is no need to assume abnormal connections between brain areas^20^. At the same time, the results show clear differences from congenital synaesthesia, and one cannot use the results to draw any conclusions regarding it. They are though in line with earlier brain-imaging and event-related-potential (ERP) results suggesting that hypnotically^1,39^ – or otherwise^3^ – suggested hallucinations can be experienced as real. In the earlier studies^1,3^, participants had around 30 seconds to create the hallucination, making it impossible to know whether or not mental imagery was used. Our results support the AST^4,26^ along with other theories assuming that hypnosis can lead to a special brain state (see e.g.^5,28,30–34,40,41^) needed to trigger hallucinations preconsciously without resort to mental imagery or other conscious strategies.

Two of the four highly hypnotizable participants reported visually experiencing inducer and concurrent simultaneously, in external space. They named according to the suggestions – at least part of the time – and showed a clear target effect in their eye movements. Their subjective experience appeared very similar to a projector-type synaesthete, with the key difference that they reported the object as having the suggested colour and not – as is typical in GCS^13^ – the colour being located somewhere above the symbol. Unlike all the other participants, including the simulators, their gaze duration did not slow down during their synaesthetic experiences, supporting their claims that they really saw the target symbols in altered colours (*cf*.^14^).

That these participants saw the inducer non-veridically clearly separates them from typical projector synaesthetes. Indeed, one of them (S1) was entirely unaware of the veridical colour. It is unlikely that any “true” synaesthete would have named the suggested colour when the instructions clearly said to name veridical colour. It is even more unlikely that they would have been unaware of the veridical colour^12,42^.

A third highly hypnotizable participant (S2) reported a strong association between the symbol and the suggested colour, but with no affect on visual experience. S2 named all symbols according to their veridical colour. Nevertheless, she showed a clear target effect, being significantly slower naming an inducer symbol by its veridical colour compared to non-targets. Both her experience and objective results fit well with accounts of by far the most typical form of synaesthesia: the *associator* type^21^. Previous research^42^ suggests that a projector-type synaesthete would have displayed this same response pattern: i.e., showing more difficulty naming the veridical colour of an inducer that is incongruent with the automatically occurring concurrent.

The experience and response pattern of the fourth participant (S4) did *not* resemble synaesthesia. She performed the tasks very similarly to ConLoG participants. The heterogeneity of HG in experience and response pattern is well in line with previous studies (e.g.^16,17^), which point out how especially highly suggestible participants can make a full response to a suggestion – or equally no response. Partial responses of various kinds are, clearly, also possible. We were able to identify all these variations.

Participants in the control groups ConLoG and ConSimG did not experience any change in perception of the inducer symbols. All participants in ConSimG were clearly slower in EC. The same effect was observed in varying degrees in ConLoG, though they still named the veridical colours. The slow down in ConSimG can be explained by the need to evaluate each symbol to decide if it was a target symbol; the colour name associated with the symbol then had to be retrieved before responding. More research is needed to understand why ConLoG found naming veridical colours in EC harder.

Use of the ConSimG control group add invaluable information: their results were homogenous and differed markedly from HG and ConLoG. When asked, all ConSimG participants spontaneously reported the strategies they used: i.e., repeating the instructions to themselves or memorizing images of the target items. Asked the same question, all participants in HG and ConLoG denied making any attempt. The results support the claim^43,44^ that hypnotized individuals behave and report their experiences honestly. They tell against the view^45^ whereby the hypnotized individual is an active problem-solving agent trying to decide what is required for responding successfully.

Our results are in line with two earlier studies^7,8^ that used hypnosis to induce GCS. They likewise found that the hypnotic suggestion created a strong association in the suggested inducer/concurrent pair for highly hypnotizable participants. This study had the advantage that subjective reports of experiences and strategies could be verified with objective behavioural data from eye tracking. What the results showed was that strategies – when used – require the equal evaluation of target and non-target symbols.

That the hypnotically induced analogue of synaesthesia differs phenomenologically from congenital synaesthesia implies that the underlying neural mechanism is different. That said, it is likely that they share certain neurological features: e.g., absorption^46^. This suggests a fruitful area for further research. Our results highlight the importance of using subjective experiences to guide selection of participants for future studies (see also^17^) – something that has generally not been recognized in other studies. The results contribute to a better understanding of hypnosis by showing that hypnotically induced hallucinations can occur fully automatically in some cases, even without conscious awareness of their hallucinatory nature. They open the door to new possibilities for increasing understanding of perception, automaticity, (see e.g.^47^) and conscious awareness.

## Methods

### Participants

All participants had normal or corrected-to-normal vision. All gave written informed consent to the experiment (including audio and video recording), described to them as a colour-naming task with eye tracking. The HG and ConLoG groups were informed that hypnosis would be used at some point. Each participant was interviewed afterwards, the full purpose of the experiment explained, and a request made for any questions concerning hypnosis or the experiment in general. Participants either received €10 as compensation or were given credits in an introductory psychology course. The research was conducted according to the ethical standards of the American Psychological Association (APA) and approved by the ethics committee of the University of Turku, Finland (Statement 18/2011).

Participants for the hypnosis groups (HG, ConLoG: *n* = 61) were selected from students in an introductory psychology course who completed the HGSHS:A^17^. Fifteen participants (seven who had an HGSHS:A score > 9 and eight who had a score < 4) were invited to further testing with a modified version of the SHSS:C:^18^ in the item “anosmia to ammonia”, strong perfume was used instead of ammonia to avoid risk of unpleasant experiences; this item is often left out entirely (see e.g.^8,48^) due to the associated risks. Two participants from this group (>9 HGSHS:A score) responded to the negative visual hallucination item and were invited to participate in the experiment. An additional two, highly hypnotizable individuals were invited to participate based on previous experience of their responsiveness to visual hallucination suggestions^23^. All those who scored <4 in HGSHS:A scored 4 or less in SHSS:C. Four were invited to participate in the experiment, with preference for (higher) age, since HG included only one participant under 30 years.

Altogether, eight persons were invited to participate in the experiment under the hypnosis conditions: four in HG (three females, one male; mean age 37, SD 12.1; HGSHS:A M = 11.5, SD 0.5; SHSS:C M = 9.75; SD 1.3) and four in ConLoG (four females; mean age 26.7, SD 8.7; HGSHS:A M = 1.5, SD 1.3; SHSS:C M = 2, SD 0.6). During presentation of the SHSS:C, participants were given a posthypnotic suggestion that later – while in a normal, baseline condition – a hypnotic condition would immediately recur when the experimenter (SK) counted to three, with a count from three to one cancelling the condition. This method of inducing and terminating hypnosis was used throughout.

A simulator control group (a modification from,^36^) was added (ConSimG) to provide better control over the demand characteristics (for details, see^36^) of the experiment. Volunteers were solicited who matched the hypnosis groups as well as possible on age and education. Eight were invited to participate in the experiment (eight females; age M = 36, SD 14.4).

### Apparatus

Eye tracking was done with a table-mounted Eyelink 1000 produced by SR Research, Canada. Eye movements were recorded binocularly, though only the data from one eye were saved. The sampling rate was 1,000 Hz. The Eyelink 1000 is an infrared video-based tracking system with hyperacuity image processing; its spatial resolution is 0.5 degrees. Stimuli were presented on a 21-inch ViewSonic G225f computer screen (refresh rate 100 Hz, resolution 1024 × 768).

### Stimuli and task

Participants viewed the screen from approximately 70 cm. Each stimulus presentation (three each under NC and NC2, six under EC) consisted of 25 geometric shapes (circles, triangles, squares, crescents, crosses, and hearts) in a 5 × 5 array. On-screen shape size varied between 2.6 × 3.2 cm and 4.2 × 3.4 cm, corresponding respectively to approximately 2 × 2.6° versus 3.4 × 2.8° visual angle. Under all three conditions, the task was to name symbol colours as quickly and accurately as possible.

**Figure 6:**
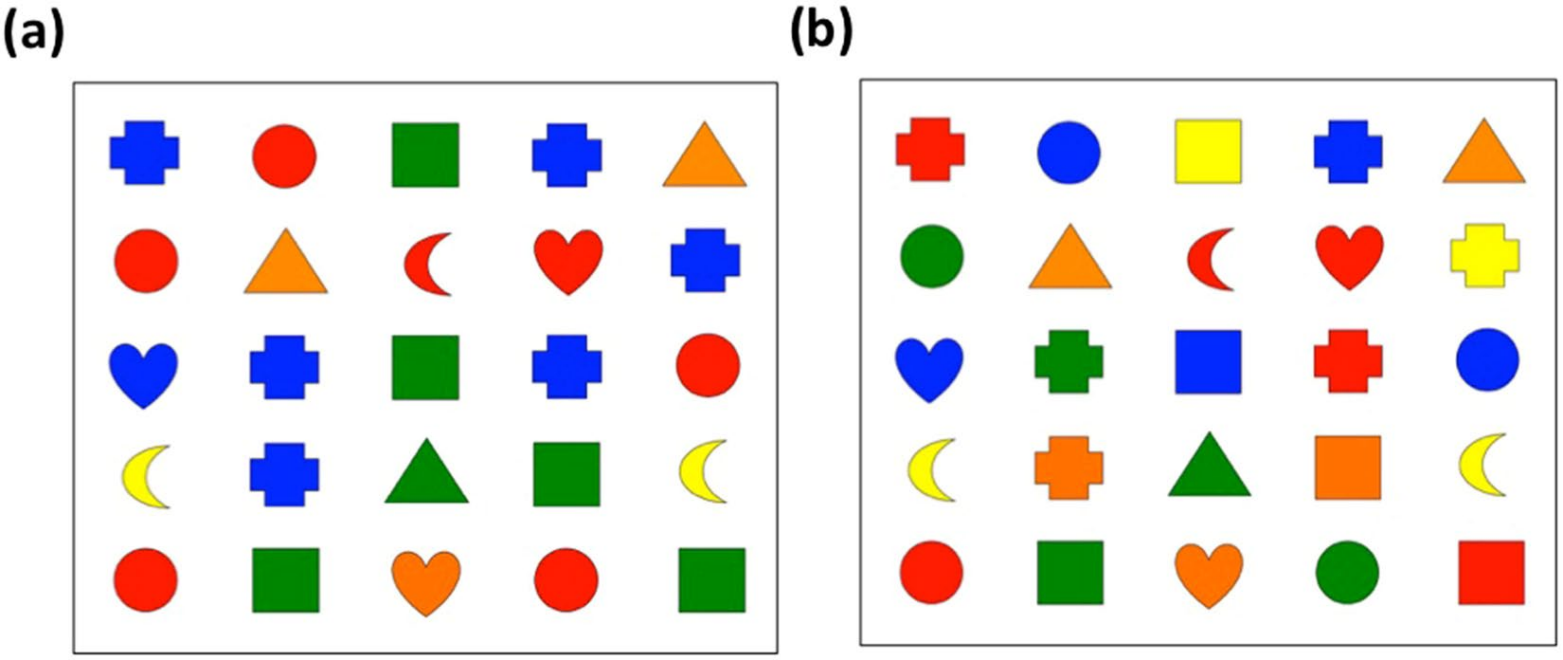
An example stimulus array (**a**) under normal conditions NC, NC2 (**b**) under experimental condition EC. If a participant should experience all the given inducer/ concurrent suggestions as true hallucinations, the arrays would look identical.

In NC and NC2, each of the crescents, hearts, and triangles were randomly assigned to be red, blue, green, orange, or yellow. The circles were always red, the squares always green, the crosses always blue (Fig. 6a). The stimuli in EC were identical, except that, while the crescents, hearts, and triangles kept the same colouring as before, each of the circles, squares, and crosses were randomly assigned one of the five colours (Fig. 6b,* cf*. Fig. 6a).

### Procedure

Two persons (JK and SK) conducted all experiments. After signing the consent form, participants sat in front of a computer screen. JK sat to the right at a distance of 1.5 m. and collected the eye-movement data. SK sat behind the participant (also at a distance of 1.5 m.) except when giving the verbal instructions, when he stood. Each participant was tested separately. Participants received on-screen instructions for the first task by pressing the space bar; they were told to read at their own pace. The instructions were to name aloud the colour of each symbol as quickly but clearly as possible, avoiding errors – beginning from the upper left and proceeding to the lower right, like reading a book. The three conditions proceeded always in the same order: NC (three array presentations), EC (six), NC2 (three). Each condition was preceded by a rehearsal array consisting of ten symbols to make sure the task was understood. A short pause to calibrate the eye-movement software was required before each presentation.

After completing the three NC arrays, participants proceeded to the six EC arrays. Prior to EC, ConSimG (*n* = 8) only received precise on-screen instructions (see Supplementary Methods 1) how to simulate the behaviour of a person experiencing hypnotically induced colour change.

The EC began with SK saying “soon I will count to three…”. Participants were then instructed to see all squares as green, all circles as red, and all crosses as blue For HG and ConLoG, these words triggered the earlier posthypnotic suggestion to induce hypnosis, whereas for ConSimG they meant only to close one’s eyes and listen carefully (note that only the suggestions were given under hypnosis, see Supplementary Methods 2). Following the on-screen instructions, SK said that it was time to continue with a second set of arrays but that these would again be preceded by a short rehearsal.

After finishing the six EC arrays and before being introduced to the final three NC2 arrays, HG and ColLoG had their colour-change suggestions cancelled by SK (Supplementary Methods 3). SK verbally instructed all groups that they would now complete one last set of arrays. For ConSimG, he added that they should do so as in the first condition: i.e., by naming the real colours.

All sessions were audio recorded to facilitate counting errors made during the naming task. All EC sessions were videotaped with the computer screen clearly visible (SK stood behind the participant to do the recording). After the experiment was over, participants were shown the video and asked about strategies employed. Participants in HG and ConLoG were additionally asked how they experienced the suggestions (if at all) and whether they perceived colour changes on the screen or only in their thoughts. The use of video-tape playback is based on the *experiential analysis technique* (EAT,^17^), which focuses on individual differences in response to hypnotic suggestions among hypnotizable individuals.

Finally, all participants were given six attempts to win €50 if they could do the EC task again but more quickly than their fastest time in NC, using a strategy we would give them: namely, to associate the green squares with a football field or the Libyan flag, the red circle with a red ball or the Japanese flag, and the blue cross with the Finnish flag. These associations were the most popular in a pilot study in which five persons – who did not take part in the subsequent study – were given the task and asked to create a memory-association strategy to remember the symbol/colour associations. They were also told they could use any strategy they came up with on their own. No one met the challenge.

### Data preparation and analysis

Trial times were determined from the audio recordings as time lapsed from naming the colour of the first shape on the screen until naming the last. The audio recordings were also used to determine the number of errors made.

Eye fixations shorter than 50 ms were either removed, or merged with a nearby fixation if the distance was < 1°. Fixations related to return sweeps (from the end of one line to the start of the next) were removed if they landed on an item other than the first. “Interest areas” were created around each geometric shape and gaze duration (i.e., summed duration of all fixations landing on the shape during first pass) computed. Only correctly responses were included in subsequent analysis, except that responses where the error corresponded with the suggestion were considered synaesthetic effects. Gaze durations > 2.5 SD from the participant’s grand mean were considered outliers and removed prior to statistical analysis.

For each participant, the critical comparison is between responses for which a synaesthetic induction was made (i.e., targets) vs. those for which none was made (non-targets). A power calculation assuming effect size *d* = 0.5, power 0.80, alpha level 5%, indicates that 64 observations are required per condition. We had 78 targets and 72 non-targets, allowing us to determine statistically significant differences between conditions.

Data analysis was conducted separately for each participant in HG and ConLoG, with linear regression using the *R* statistical software. For trial times, a dummy coded task (NC, EC, NC2; NC as baseline) was entered as predictor. For gaze duration, effect-coded item type (non-target = −1, target = 1), dummy coded task (NC, EC, NC2; NC as baseline), and item type *task-interaction term were entered as predictors. The effects of item type were examined by fitting the model at different task levels. For the control group data, a linear mixed-effects model was computed using the *lme4* package (see^49^). Item type and task were entered as fixed effects and participants as a random factor. The results were interpreted by examining the model estimates and their 95% CIs^50^.

### Data Availability

The relevant datasets are available from the corresponding author on request.

## Electronic supplementary material

Supplementary Information
