# Supplementary material for: Synaesthesia-type associations and perceptual changes induced by hypnotic suggestion
Source: Sci Rep. 2017 Dec 11;7:17310. doi: 10.1038/s41598-017-16174-y (PMC5725458; doi:10.1038/s41598-017-16174-y)
Supplement: Supplementary file 1 — Supplementary Information [file 41598_2017_16174_MOESM1_ESM.pdf]

SUPPLEMENTARY METHODS

Synaesthesia-type associations and perceptual changes induced by hypnotic suggestion

Sakari Kallio, Mika Koivisto & Johanna K. Kaakinen

1. Instructions given to the simulator control group ConSimG before EC on how to simulate a person experiencing hypnotic colour hallucinations.

Your next task is to simulate a person who has been given a hypnotic suggestion to experience certain symbols as ***always*** having a certain color. An example could be that a star symbol is always seen as red. When this person sees a star she always says that it is red. Your task, then, is to name the symbol colours as quickly and accurately as possible (as previously), except to say "red" if you see a star symbol. Try to do the task in such a way that, if anyone were to analyse the results, it would not be possible to say whether you had experienced the colours as instructed or simply pretended to. You can use any strategies you can think of, to succeed as well as possible. In a minute, you will get exact instructions how to do this task.

2. Instructions given in EC to all three groups.

Soon I will count to three. After that you should close your eyes and listen very carefully to what I tell you. Ready? One, two, three.... You will do the same task as before. In these arrays, however, squares are always green, circles always red, and crosses always blue. Now you can open your eyes and look at the screen in front of you. (*On the screen is a picture of a green square, a red circle, and a blue cross, arranged vertically.*) When you shortly begin the task, you will see only green squares, red circles, and blue crosses – just like the ones in front of you. There will be other symbols in the array, and they can be of different colours. It is possible that you will forget all that I have told you here, but nevertheless you only see green squares, red circles and blue crosses, just as you see now, along with other symbols, which can be of any colour. The task is easy, and you will see all the symbols and colours clearly. Good! Three, two, one....

Note: All participants performed the EC task in in their normal baseline state of consciousness. Only the suggestions for colour change were given under hypnosis (as were the instructions to cancel those suggestions), and only for HG and ConLoG.

3. Cancelling the suggestions for HG and ConLoG after the EC.

Please close your eyes and listen carefully. Good! One, two, three.... From now on, any of the symbols you see on the screen can have any of the five colours. Squares can be any colour, circles can be of any colour, crosses can be of any colour. You can see the colour of each symbol easily. Three, two, one....
